# PET Imaging of the Adenosine A_2A_ Receptor in the Rotenone-Based Mouse Model of Parkinson’s Disease with [^18^F]FESCH Synthesized by a Simplified Two-Step One-Pot Radiolabeling Strategy

**DOI:** 10.3390/molecules25071633

**Published:** 2020-04-02

**Authors:** Susann Schröder, Thu Hang Lai, Magali Toussaint, Mathias Kranz, Alexandra Chovsepian, Qi Shang, Sladjana Dukić-Stefanović, Winnie Deuther-Conrad, Rodrigo Teodoro, Barbara Wenzel, Rareş-Petru Moldovan, Francisco Pan-Montojo, Peter Brust

**Affiliations:** 1 ROTOP Pharmaka Ltd., Department of Research and Development, Dresden 01328, Germany; 2 Helmholtz-Zentrum Dresden-Rossendorf (HZDR), Department of Neuroradiopharmaceuticals, Institute of Radiopharmaceutical Cancer Research, Research Site Leipzig, Leipzig 04318, Germany; t.lai@hzdr.de (T.H.L.); m.toussaint@hzdr.de (M.T.); s.dukic-stefanovic@hzdr.de (S.D.-S.); w.deuther-conrad@hzdr.de (W.D.-C.); r.teodoro@hzdr.de (R.T.); b.wenzel@hzdr.de (B.W.); r.moldovan@hzdr.de (R.-P.M.); p.brust@HZDR.de (P.B.); 3 PET Imaging Center, University Hospital of North Norway (UNN), Tromsø 9009, Norway; mathias.kranz@uit.no; 4 Nuclear Medicine and Radiation Biology Research Group, The Arctic University of Norway, Tromsø 9009, Norway; 5 University Hospital Munich, Department of Psychiatry, Ludwig-Maximilians-Universität (LMU) Munich, Munich 80336, Germany; alexandra.chovsepian@med.uni-muenchen.de (A.C.); Francisco.Pan-Montojo@med.uni-muenchen.de (F.P.-M.); 6 University Hospital Munich, Department of Neurology, Ludwig-Maximilians-Universität (LMU) Munich, Munich 81377, Germany; shangqi0911@gmail.com; 7 University Hospital Carl Gustav Carus, Clinic of Neurology, Technische Universität Dresden (TUD), Dresden 01307, Germany

**Keywords:** adenosine A_2A_ receptor, Parkinson’s disease, rotenone-based mouse model, PET imaging, [^18^F]**FESCH**, two-step one-pot radiosynthesis

## Abstract

The adenosine A_2A_ receptor (A_2A_R) is regarded as a particularly appropriate target for non-dopaminergic treatment of Parkinson’s disease (PD). An increased A_2A_R availability has been found in the human striatum at early stages of PD and in patients with PD and dyskinesias. The aim of this small animal positron emission tomography/magnetic resonance (PET/MR) imaging study was to investigate whether rotenone-treated mice reflect the aspect of striatal A_2A_R upregulation in PD. For that purpose, we selected the known A_2A_R-specific radiotracer [^18^F]**FESCH** and developed a simplified two-step one-pot radiosynthesis. PET images showed a high uptake of [^18^F]**FESCH** in the mouse striatum. Concomitantly, metabolism studies with [^18^F]**FESCH** revealed the presence of a brain-penetrant radiometabolite. In rotenone-treated mice, a slightly higher striatal A_2A_R binding of [^18^F]**FESCH** was found. Nonetheless, the correlation between the increased A_2A_R levels within the proposed PD animal model remains to be further investigated.

## 1. Introduction

Parkinson’s disease (PD) is characterized by the degeneration of dopaminergic neurons in the brain, especially in the substantia nigra, resulting in a decreased dopamine level in the striatum. Consequently, stimulation of the dopamine D_2_ receptor (D_2_R) is reduced and, further, the morphology of striatal neurons and synaptic connections is changing. These effects lead to motor dysfunctions like bradykinesia, tremor, rigor, and postural instability [1,2]. The standard therapy of PD is based on the pharmacological increase of dopamine levels either by administration of L-DOPA as metabolic precursor of dopamine or by dopamine agonists. Further strategies to raise or maintain the dopamine levels are based on inhibitors for dopamine reuptake and dopamine-degrading enzymes (e.g., monoamine oxidase B, catechol-*O*-methyl transferase) [2]. These dopamine- increasing drugs have only a positive effect on motor functions at short-term treatment but cause undesirable side effects at long-term treatment like dyskinesia, nausea, and hallucinations. Due to these limitations, the medicinal research is currently focusing on non-dopaminergic therapies of PD [2,3].

The adenosine A_2A_ receptor (A_2A_R) is a G-protein coupled receptor that is highly expressed in the basal ganglia of the brain, mainly in striatopallidal GABAergic neurons. Furthermore, in this brain area the A_2A_R and the D_2_R are co-localized and form heteromeric receptor complexes while showing contradictory effects on motor functions [2]. For example, an L-DOPA-induced increase of dopamine stimulates the D_2_R leading to enhanced motor activity. Activation of the A_2A_R by adenosine or appropriate agonists results in the inhibition of the D_2_R and, thus, in reduced dopamine binding [2,4,5]. Pharmacological inhibition of the A_2A_R amplifies the D_2_R-dependent signaling cascade and improves motor symptoms [2,3,5,6]. Since the A_2A_R modulates the dopamine binding affinity of the D_2_R, selective A_2A_R antagonists have great potential as appropriate non-dopaminergic PD therapeutics [2,3,6]. In clinical phase II and III trials, the A_2A_R antagonists **istradefylline**, **preladenant**, and **tozadenant** improved motor symptoms in PD patients [7,8,9]. Out of these, **istradefylline** (Nourianz^®^) has already been approved in Japan [10] and currently by the U.S. Food and Drug Administration (FDA) as an adjunctive treatment to L-DOPA.

Furthermore, a comparative neuropathological postmortem study on brain slices from healthy subjects and PD patients showed a 2.5-fold increased striatal A_2A_R protein level already at early PD stages pointing out that A_2A_R upregulation is an initial event in the pathogenesis of the disease [11]. Though, it remains unclear whether there is a correlation between altered A_2A_R expression and motor symptoms in PD.

Molecular imaging of the A_2A_R with positron emission tomography (PET) enables the non-invasive investigation of pathological changes of the receptor level in the human brain [12].

**Figure 1:**
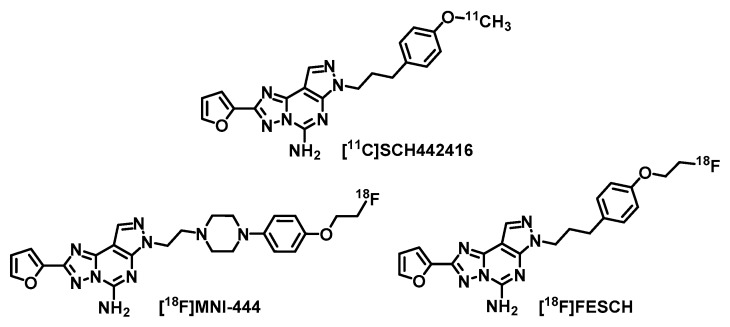
Molecular structures of the herein discussed radiotracers for positron emission tomography (PET) imaging of the adenosine A_2A_ receptor (A_2A_R).

A clinical PET study using [^11^C]**SCH442416** (Figure 1) revealed a 70–80% increased A_2A_R availability in the striatum of PD patients with L-DOPA-induced dyskinesia [13]. This strongly indicates a key role of A_2A_R not only in the development of PD [11] but also in the progression of the disease and in association with the dopaminergic standard therapy [2,13]. Hence, A_2A_R-PET is considered as an appropriate tool for early diagnosis and staging of PD as well as evaluation of potential A_2A_R antagonists for PD treatment by means of receptor occupancy studies. The main disadvantage of ^11^C-labeled radiotracers for clinical routine applications is the short half-life of the nuclide (*t*_1/2_ = 20.3 min). To date, there is only one ^18^F-labeled (*t*_1/2_ = 109.7 min) A_2A_R radiotracer, [^18^F]**MNI-444** [14,15] (Figure 1), that has already been evaluated in a clinical PET study. In healthy volunteers [15], a high uptake of activity in the striatum has been observed. However, no follow-up studies have been published since 2015 and further data of [^18^F]**MNI-444** regarding specificity of A_2A_R binding in humans or selectivity over the adenosine A_1_ receptor (A_1_R), which is also highly expressed in the brain [16,17], are not yet available. For that reason, we selected the well-studied and highly A_2A_R-selective radiotracer [^18^F]**FESCH** [18,19,20,21] (*K*_i_(*h*A_2A_R) = 12.4 nM, *K*_i_(*h*A_1_R) ~ 10 µM, former [^18^F]**MRS5425** [18]) for our purposes, which is the [^18^F]fluoroethoxy analog of [^11^C]**SCH442416** (Figure 1). [^18^F]**FESCH** has been evaluated in healthy rats and in rats with unilateral PD symptoms by PET showing an A_2A_R-specific binding in the striatum and a significantly increased A_2A_R-mediated uptake in the 6-hydroxydopamine-lesioned hemisphere of 9–12% [19,21]. Therefore, [^18^F]**FESCH** has been stated as the most suitable radiotracer for quantification of the A_2A_R in the brain with PET [21].

The rotenone-based mouse model has been well established for pre-clinical investigations of PD [22,23,24]. Chronic administration of the neurotoxin rotenone to mice leads to relapse of dopaminergic neurons and changes in motor functions similar to the symptoms in PD patients [24,25,26]. In this small animal PET/MR imaging study with [^18^F]**FESCH**, we aimed to validate if rotenone-treated mice reflect the aspect of striatal A_2A_R upregulation in PD. In addition, we investigated [^18^F]**FESCH** by in vitro autoradiography on mouse brain slices and in vivo metabolism as well as baseline and blocking PET studies in healthy CD-1 mice.

## 2. Results and Discussion

The A_2A_R antagonist **FESCH** and the required known phenol precursor desmethyl SCH442416 for ^18^F-labeling were synthesized in three or two steps, respectively, from commercially available 2-(furan-2-yl)-7*H*-pyrazolo[4,3-*e*][1,2,4]triazolo[1,5-*c*]pyrimidine-5-amin and 1-(3-bromopropyl)-4- methoxybenzene according to procedures described in the literature [18,27,28]. Evaluation of **FESCH** in an established binding assay revealed high A_2A_R affinity (*K*_i_(*h*A_2A_R) = 0.6 ± 0.1 nM, n = 4) and good A_1_R selectivity (*K*_i_(*h*A_1_R) = 203 ± 40 nM, n = 4).

**Scheme 1:**
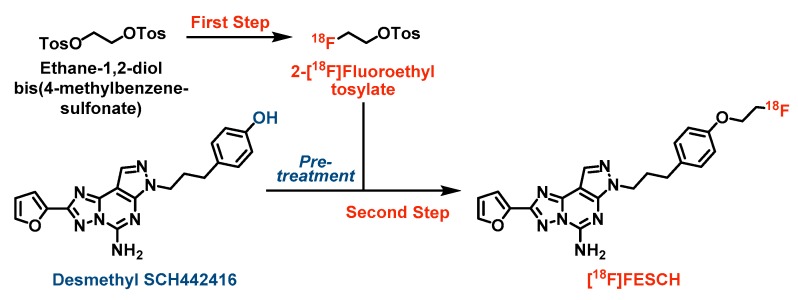
Simplified two-step one-pot radiosynthesis of [^18^F]**FESCH**. Reagents and conditions: (First step) 2 mg ethane-1,2-diol bis(4-methylbenzenesulfonate) in 100 µL MeCN, ~ 3 GBq azeotropically dried K^+^/[^18^F]F^-^/K_222_-carbonate complex in 400 µL MeCN, 90 °C, 10 min; (Second step) 2.5 mg desmethyl SCH442416 pre-treated with 10 µL TBAOH_aq._ (40%) in 490 µL MeCN at 90 °C for 10 min, directly added to 2-[^18^F]fluoroethyl tosylate, 120 °C, 10 min.

The initially published radiosynthesis of [^18^F]**FESCH** comprises a two-step two-pot procedure [19,20]. Briefly, the respective [^18^F]fluoroethyl intermediate from the ^18^F-labeling step was isolated either by semi-preparative HPLC [19] or solid-phase extraction on a cartridge [20] and, only then, reacted with the phenol precursor desmethyl SCH442416. To simplify the radiosynthesis and avoid loss of activity through purification of the [^18^F]fluoroethyl intermediate, we developed a two-step one-pot strategy for the production of [^18^F]**FESCH** [29] (see Scheme 1). First, ethane-1,2-diol bis(4-methylbenzenesulfonate) was ^18^F-labeled via nucleophilic substitution of one tosylate group using anhydrous K^+^/[^18^F]F^-^/K_222_-carbonate complex in acetonitrile. Notably, desmethyl SCH442416 was pre-treated with aqueous tetrabutylammonium hydroxide (TBAOH) in acetonitrile to generate the activated phenolate which was directly reacted with the non-isolated 2-[^18^F]fluoroethyl tosylate. Besides, further bases and solvents were tested for the [^18^F]fluoroethylation of desmethyl SCH442416 resulting in decreased yields of [^18^F]**FESCH** mainly due to partial decomposition of the phenol precursor under these conditions. Preliminary experiments using potassium carbonate or cesium carbonate (1.5–2.5 eq.) for activation of desmethyl SCH442416 (1–2 mg) and either acetonitrile or a 1:1 mixture of acetonitrile and *N*,*N*-dimethylformamide or *tert*-butanol as solvents revealed labeling yields of 4–14% based on TLC analysis of the reaction mixtures after 10 min at 120 °C, respectively. Replacement of the base by TBAOH (2.5 eq.) and the use of acetonitrile only resulted in a significantly increased labeling yield of 25%. Final optimization was achieved by increasing the amount of the phenol precursor to 2.5 mg leading to 46.4 ± 8.5% (n = 9) of non-isolated [^18^F]**FESCH**.

**Figure 2:**
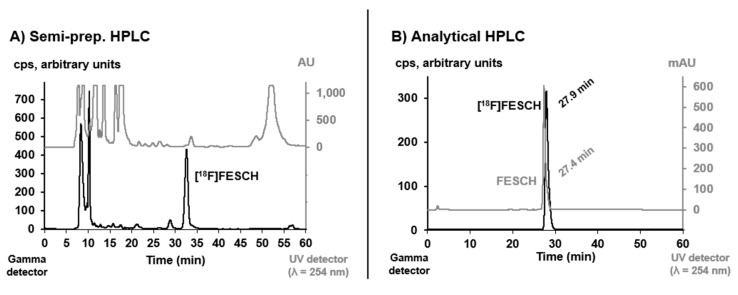
(**A**) Semi-preparative HPLC profile of the crude reaction mixture for isolation of [^18^F]**FESCH** (column: Reprosil-Pur 120 C18-AQ, 250 × 10 mm, particle size: 10 µm, eluent: 50% MeCN/20 mM NH_4_OAc_aq._, flow: 7 mL/min). (**B**) Analytical HPLC profile of the formulated radiotracer [^18^F]**FESCH** spiked with the non-radioactive reference **FESCH** (column: Reprosil-Pur 120 C18-AQ, 250 × 4.6 mm, particle size: 5 µm; eluent: 26-90-26% MeCN/20 mM NH_4_OAc_aq._, flow: 1 mL/min).

[^18^F]**FESCH** was purified by semi-preparative HPLC (see Figure 2) and concentrated via solid-phase extraction on a pre-conditioned reversed-phase cartridge followed by elution with absolute ethanol. After evaporation of the solvent, the radiotracer was finally formulated in sterile isotonic saline with a maximum ethanol content of 10% for better solubility. The identity of [^18^F]**FESCH** was confirmed by analytical HPLC using an aliquot of the final product spiked with the non-radioactive reference compound (Figure 2).

The herein described one-pot radiolabeling strategy provided [^18^F]**FESCH** with a high molar activity of 116 ± 18.5 GBq/μmol (n = 7, end of synthesis) and an overall radiochemical yield of 16.1 ± 1.5% (n = 9, end of bombardment), which is a significant improvement compared to the published two-pot procedure (7 ± 2% [20]). The required total synthesis time of 114 ± 6 min and the achieved radiochemical purity of ≥ 98% were very similar for both methods.

In vitro stability of [^18^F]**FESCH** was examined in isotonic saline and pig plasma. Samples of each medium were analyzed by radio-HPLC after 60 min incubation at 37 °C and no degradation or defluorination of the radiotracer was observed. This result is contradictory to the published stability of [^18^F]**FESCH** especially in saline where only 85–90% of intact radiotracer have been detected by radio-TLC while multiple spots were observed [20]. Thus, Khanapur et al. [20] formulated [^18^F]**FESCH** in phosphate-buffered saline, but Bhattacharjee et al. [19] used a saline solution of [^18^F]**FESCH** for biological investigations. At present, we have no explanation for that and as we detected only the intact radiotracer in isotonic saline (see Figure 2), formulation of [^18^F]**FESCH** in another medium was not required.

The distribution coefficient of [^18^F]**FESCH** was determined by partitioning between *n*-octanol and phosphate-buffered saline (PBS, pH = 7.4) at ambient temperature using the conventional shake-flask method. The obtained logD_7.4_ value of 1.97 ± 0.17 (n = 4) emphasizes the lipophilic character of the radiotracer which allows a passive diffusion through the blood–brain barrier [30,31,32]. However, there is a discrepancy compared to the published logD value of 3.16 ± 0.03 [20] which might be caused by different experimental setups. 

**Figure 3:**
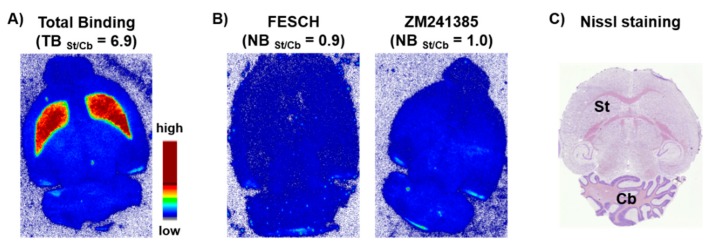
Representative autoradiographic images of transversal CD-1 mouse brain slices. (**A**) In vitro distribution of activity after incubation with 0.2 MBq/mL of [^18^F]**FESCH** (TB = total binding). (**B**) Non-specific binding (NB) of [^18^F]**FESCH** determined in the presence of 1 μM of **FESCH** or **ZM241385**, respectively, as blocking agents. (**C**) Nissl staining, St = Striatum, Cb = Cerebellum.

In vitro autoradiography studies were accomplished by incubating sections of mouse brain with [^18^F]**FESCH**. Non-specific binding was assessed by co-incubation with an excess of either **FESCH** or **ZM241385**, respectively. The images demonstrated an A_2A_R-specific accumulation of [^18^F]**FESCH** in the striatum (Figure 3), which is characterized by the binding parameters *K*_D_ = 4.69 ± 1.17 nM and *B*_max_ = 497 ± 97 fmol/mg wet weight consistent with the literature [33].

**Figure 4:**
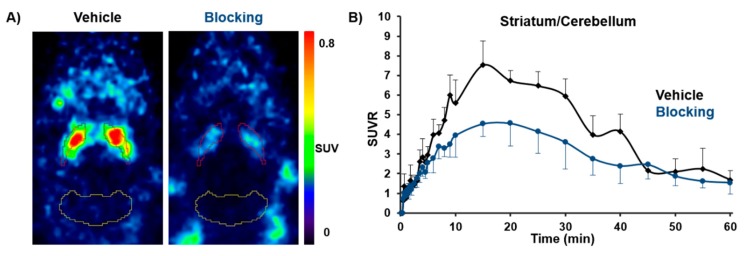
(**A**) Representative horizontal PET images of [^18^F]**FESCH** uptake (average 10–30 min) in the brain of healthy CD-1 mice under vehicle (15 min pre-injection of DMSO:Kolliphor^®^ EL:0.9% NaCl, 1:2:7, 5 µL/g) and blocking conditions (15 min pre-injection of **tozadenant** 2.5 mg/kg in DMSO:Kolliphor^®^ EL:0.9% NaCl, 1:2:7, 5 µL/g; red = striatum; yellow = cerebellum). (**B**) Averaged TACs of [^18^F]**FESCH** for vehicle (n = 4) and **tozadenant** pre-injected mice (n = 4) with SUVRs for striatum over cerebellum.

For in vivo imaging investigations in healthy CD-1 mice (n = 12, 10 weeks, 30–35 g), [^18^F]**FESCH** was injected intravenously (baseline: 2.8 ± 2 MBq, vehicle: 5.8 ± 2 MBq, blocking: 6.4 ± 2.5 MBq with n = 4, respectively) and whole body scans were performed for 60 min in listmode with a Mediso nanoScan^®^ PET/MR scanner followed by dynamic reconstruction. Time-activity curves (TACs) were generated for regions of interest such as striatum and cerebellum as reference region. The obtained PET images showed a high striatal uptake of [^18^F]**FESCH** with a standardized uptake value ratio (SUVR) for striatum over cerebellum of > 5 (9–30 min post injection (p.i.)). In vivo selectivity for the A_2A_R was highlighted by the significant reduction of the SUVR by 29–42% (9–30 min p.i. with *p* < 0.05) after pre-injection of **tozadenant** as blocking agent (Figure 4). The highest achievable dose of **tozadenant** was 2.5 mg/kg with respect to solubility and volume of injection as well as the concentrations of DMSO and Kolliphor^®^ EL suitable for in vivo application in mice. The herein observed blocking effect is in accordance with the estimated A_2A_R occupancy for **tozadenant** in rhesus monkey of about 72% at 5 mg/kg [14]. These results indicate that [^18^F]**FESCH** is a promising radiotracer for molecular imaging of the A_2A_R in the brain.

**Figure 5:**
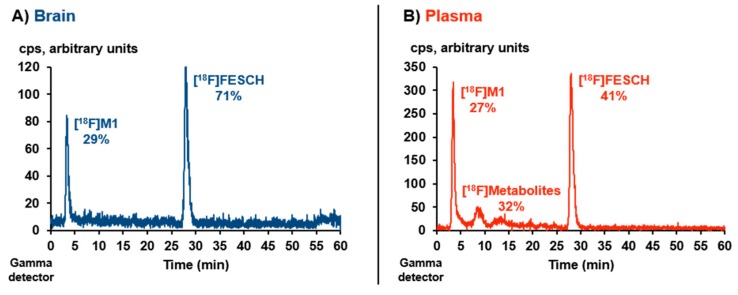
Representative in vivo metabolism study of CD-1 mouse plasma and brain samples at 15 min p.i. of [^18^F]**FESCH** (~ 17 MBq): Analytical radio-HPLC profiles of extracted (**A**) brain and (**B**) plasma sample (column: Reprosil-Pur 120 C18-AQ, 250 × 4.6 mm, particle size: 5 µm; eluent: 26-90-26% MeCN/20 mM NH_4_OAc_aq._, flow: 1 mL/min).

However, a representative metabolism study revealed only moderate in vivo stability of [^18^F]**FESCH**. Analytical radio-HPLC (Figure 5) of the extracted mouse plasma sample showed 41% of intact radiotracer at 15 min p.i. (recovery of total activity = 84%). In the analyzed brain sample, one polar radiometabolite ([^18^F]**M1**) was detected accounting for 29% of the total extracted activity at 15 min p.i. (recovery = 98%).

Compared to the published study in Wistar-Unilever rats (46% intact radiotracer in plasma at 60 min p.i. [20]) the in vivo degradation of [^18^F]**FESCH** appears to be somewhat faster in mice. Notably and to the best of our knowledge, the formation of brain-penetrating radiometabolites of [^18^F]**FESCH** has not been regarded before. Based on our experiences with radiotracers bearing a [^18^F]fluoroethoxy moiety [34,35], the herein observed radiometabolite [^18^F]**M1** is proposed to be 2-[^18^F]fluoroethanol or the oxidized 2-[^18^F]fluoroacetaldehyde and 2-[^18^F]fluoroacetate, resulting from a cytochrome P450 enzyme-induced metabolic degradation [36,37], which are able to cross the blood–brain barrier [38,39,40,41].

**Figure 6:**
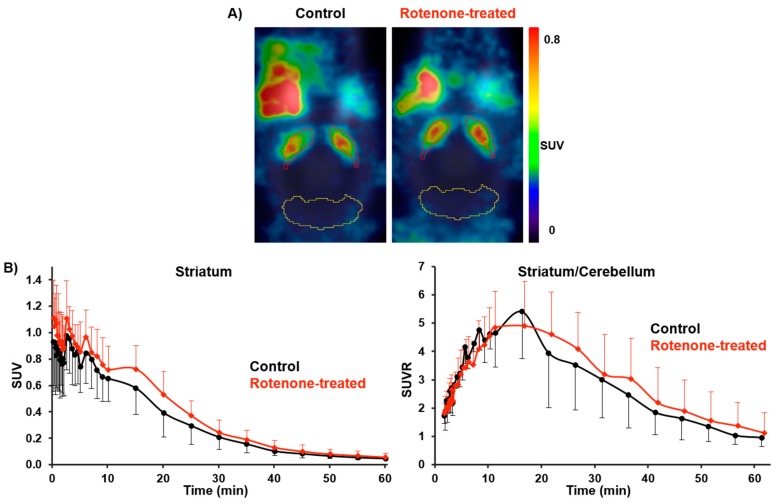
(**A**) Representative horizontal PET images of [^18^F]**FESCH** uptake (average 2–61 min) in the brain of control and rotenone-treated C57BL/6JRj mice (red = striatum; yellow = cerebellum). (**B**) Averaged TACs of [^18^F]**FESCH** for control (n = 5) and rotenone-treated mice (n = 7) with SUVs for striatum and SUVRs for striatum over cerebellum.

For PET/MR studies with [^18^F]**FESCH** in the rotenone-based mouse model of PD, the radiotracer (9.7 ± 1.3 MBq) was administrated to C57BL/6JRj mice (control: n = 5; rotenone-treated: n = 7; 16 months, 28–35 g) followed by the same imaging protocol used for the baseline and blocking studies in CD-1 mice. Although statistically not significant, the averaged TACs between 2 and 61 min p.i. revealed a slightly higher uptake of [^18^F]**FESCH** in the striatum of rotenone-treated mice compared to controls. An increase of the SUVR for striatum over cerebellum by 15–33% was observed, which was caused by the elevated SUV for striatum of 11–27% (21–61 min p.i., respectively; Figure 6).

**Figure 7:**
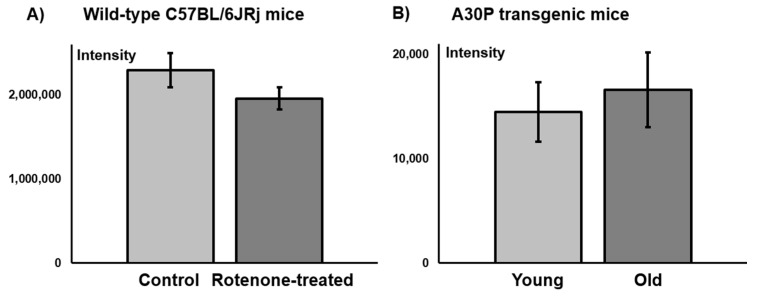
Mean fluorescence intensity of A_2A_R staining on the striatum of coronal C57BL/6JRj mouse brain sections: (**A**) Control vs. rotenone-treated wild-type mice (n = 3, respectively); (**B**) Young (39 weeks) vs. old (71–76 weeks) A30P transgenic mice (n = 3, respectively).

These results are in accordance with the determined A_2A_R levels on C57BL/6JRj mouse brain sections from a comparative in vitro immunofluorescence study. No significant difference in the fluorescence signal of the A_2A_R between control and rotenone-treated mice was observed (Figure 7).

The increased A_2A_R availability in the striatum of PD patients appears to be related to L-DOPA-induced dyskinesia. In the rotenone-treated mice, we did not identify any signs of dyskinesia and, thus, it is not surprising that in this PD mouse model no significant increase in the A_2A_R density was detected either by PET imaging or by immunofluorescence staining. With regard to dyskinesia, the rotenone model is comparable to another neurotoxin-based PD mouse model using 1-methyl-4-phenyl-1,2,3,6-tetrahydropyridine (MPTP), where only weak signs of dyskinesia were observed and this exclusively under L-DOPA treatment [42]. To date, unilateral injection of 6-hydroxydopamine (6-OHDA) is the only effective neurotoxin-related approach to replicate evidently some forms of dyskinesia in rats and mice. In the 6-OHDA mouse model, dyskinesia is detected as abnormal involuntary movements (AIMs) with a more simplified range than in rats, presenting more prominent rotational locomotion with less dystonic features, i.e., axial AIMs [42]. None of the above-mentioned AIMs were detected in the rotenone-treated mice. Consequently, it remains to be examined whether treatment with L-DOPA would lead to dyskinesia in the rotenone model as shown for the MPTP model and if this would cause a quantifiable increase in striatal A_2A_R levels. 

In contrast to the above-discussed neurotoxin-based PD animal models, the A30P mouse line represents a transgenic model of PD. This mouse line overexpresses the human A30P mutation in the α-synuclein gene, where the 30th amino acid residue alanine is replaced by proline, in all neurons. The A30P mutation is associated with rare familial cases of PD. Since α-synuclein is a main constituent of Lewy bodies, the A30P transgenic mice are characterized by Lewy body formation in neuronal cell bodies and neurites throughout the brain [43]. The α-synuclein pathology and PD-associated symptoms become prominent at about 69 weeks of age [43,44]. In an ongoing study, we detected slightly, but due to the rather small and highly variable data set statistically non-significant, higher striatal mean fluorescence intensities of the A_2A_R signal in older symptomatic A30P transgenic mice compared to younger ones without symptoms (see Figure 7). We will further investigate this PD mouse model by small animal PET/MR imaging studies using a novel derivative of [^18^F]**FESCH** currently under development.

## 3. Summary and Conclusions

In this study, we investigated the suitability of the known radiotracer [^18^F]**FESCH** for in vitro and in vivo imaging of the A_2A_R in the mouse brain by autoradiography and small animal PET/MR. Contrary to previous estimations, the herein observed brain-penetrating radiometabolite might limit the applicability of [^18^F]**FESCH** for valid quantification of A_2A_R levels in the striatum with PET. One promising strategy to decelerate the degradation of ^18^F-labeled radiotracers is the deuterium-hydrogen exchange at the metabolic labile position in the molecular structure of the compound [45]. Thus, our recent efforts are focused on a deuterated derivative of [^18^F]**FESCH** with enhanced in vivo stability particularly at the [^18^F]fluoroethoxy side chain.

In the investigated rotenone-based mouse model of PD, no significant difference in the striatal A_2A_R density between rotenone-treated mice and controls was detectable by PET imaging or immunofluorescence staining. These results indicate that the rotenone model does not reflect upregulation of striatal A_2A_R in PD, which appears to be related to dyskinesia. Therefore, it remains to be further examined whether treatment with L-DOPA could cause a detectable increase in A_2A_R levels in the striatum, which would induce observable dyskinesia in this PD model. In a parallel and ongoing study to characterize the A30P mouse model, initial findings reveal that this transgenic model might be more suitable with regard to displaying PD-like pathological changes in A_2A_R availability.

## 4. Materials and Methods

### 4.1. General Information

Chemicals and solvents were purchased from standard commercial sources in analytical grade and were used without further purification. Radio-TLCs were performed on pre-coated silica gel plates (Alugram^®^ Xtra SIL G/UV_254_, Polygram^®^ SIL G/UV_254_; Carl Roth, Karlsruhe, Germany). The compounds were localized at 254 nm (UV lamp). Radio-TLC was recorded using a bioimaging analyzer system (BAS-1800 II, Fuji Photo Film, Co. Ltd., Tokyo, Japan) and images were evaluated with Aida 2.31 software (raytest Isotopenmessgeräte GmbH, Straubenhardt, Germany). Column chromatography was conducted on silica gel (0.06–0.20 mm; Carl Roth, Karlsruhe, Germany). HPLC separations were performed on JASCO systems equipped with UV detectors from JASCO Deutschland GmbH (Pfungstadt, Germany) and activity detectors from raytest Isotopenmessgeräte GmbH (GABI Star, Straubenhardt, Germany).

Semi-preparative HPLC conditions were: Column: Reprosil-Pur C18-AQ, 250 × 10 mm; particle size: 10 µm; eluent: 50% MeCN/20 mM NH_4_OAc_aq._; flow: 7 mL/min; ambient temperature; UV detection at 254 nm.

Analytical HPLC conditions were: Column: Reprosil-Pur C18-AQ, 250 × 4.6 mm; particle size: 5 µm; gradient: 0–10 min: 26% MeCN, 10–35 min: 26% → 90% MeCN, 35–45 min: 90% MeCN, 45–50 min: 90% → 26% MeCN, 50–60 min: 26% MeCN/20 mM NH_4_OAc_aq._; isocratic: 40% MeCN/20 mM NH_4_OAc_aq._; flow: 1 mL/min; ambient temperature; UV detection at 254 nm. Molar activity was determined on the base of a calibration curve (0.05–2.0 µg **FESCH**) carried out under isocratic HPLC conditions (36% MeCN/20 mM NH_4_OAc_aq._) using chromatograms obtained at 264 nm as the maximum of UV absorbance.

No-carrier-added (n.c.a.) [^18^F]fluoride (*t*_1/2_ = 109.8 min) was produced via the [^18^O(p,n)^18^F] nuclear reaction by irradiation of [^18^O]H_2_O (Hyox 18 enriched water, Rotem Industries Ltd, Arava, Israel) on a Cyclone^®^18/9 (IBA RadioPharma Solutions, Louvain-la-Neuve, Belgium) with fixed energy proton beam using Nirta^®^ [^18^F]fluoride XL target.

### 4.2. Radiosynthesis

The aqueous solution of no-carrier-added [^18^F]fluoride (~ 3 GBq) was trapped on a Chromafix^®^ 30 PS-HCO_3_^−^ cartridge (MACHEREY-NAGEL GmbH & Co. KG, Düren, Germany). The activity was eluted with 300 µL of an aqueous K_2_CO_3_-solution (1.78 mg, 12.9 µmol) into a 4-mL V-vial containing Kryptofix 2.2.2 (K_2.2.2_, 5.6 mg, 14.9 µmol) in 1 mL MeCN. The K^+^/[^18^F]F^−^/K_2.2.2_-carbonate complex was azeotropically dried under vacuum and argon flow within 7–10 min using a Discover PETwave Microwave CEM^®^ (75 W, 50–60 °C, power cycling mode, CEM GmbH, Kamp-Lintfort, Germany). Two aliquots of MeCN (2 × 1 mL) were added during the drying procedure and the final complex was dissolved in 400 µL MeCN ready for radiolabeling.

The aliphatic radiolabeling of ethane-1,2-diol bis(4-methylbenzenesulfonate) (Sigma-Aldrich, Munich, Germany; 2 mg in 100 µL MeCN) was performed under conventional heating at 90 °C for 10 min. After pre-heating of the phenol precursor desmethyl SCH442416 (2.5 mg in 490 µL MeCN) with 10 µL TBAOH_aq._ (40%) at 90 °C for 10 min, this solution was directly added to the crude 2-[^18^F]fluoroethyl tosylate and the reaction mixture was stirred for 10 min at 120 °C. Aliquots of the reaction mixtures from both steps were analyzed by radio-TLC (EtOAc/petroleum ether; first step = 1:1; second step = 6:1) or radio-HPLC (isocratic mode, see General Information) to determine the radiochemical yields of crude 2-[^18^F]fluoroethyl tosylate (*R*_f_ = 0.7, 87.0 ± 2.3%; n = 9) and [^18^F]**FESCH** (*R*_f_ = 0.5, 46.4 ± 8.5%; *t*_R_ = 29.4 min, 34.4 ± 4.1%, decay corrected for *t*_R_ = 0 min; n = 9), respectively.

After dilution with water (1:1), the crude reaction mixture was applied to an isocratic semi-preparative HPLC (see General Information) for isolation of the desired radiotracer [^18^F]**FESCH** (*t*_R_ = 32–33 min). The collected fractions were diluted with water (total volume = 40 mL), passed through a Sep-Pak^®^ C18 Plus cartridge (Waters, Milford, MA, USA; pre-conditioned with 5 mL of absolute EtOH and 60 mL water), and eluted with 1.5 mL of absolute EtOH. Evaporation of the solvent at 70 °C under a gentle argon stream and subsequent formulation of the radiotracer in sterile isotonic saline containing 10% EtOH afforded a [^18^F]**FESCH** solution usable for biological investigations.

The identity of the radiotracer was proved by analytical radio-HPLC (see General Information) of samples of [^18^F]**FESCH** spiked with the non-radioactive reference compound **FESCH** using a gradient and an isocratic mode.

### 4.3. Investigation of In Vitro Stability and Lipophilicity (logD_7.4_)

The in vitro stability of [^18^F]**FESCH** (4–6 MBq) was studied by incubation in isotonic saline and pig plasma (500 µL each) at 37 °C. Samples were taken at 60 min and analyzed by radio-HPLC (see General Information). 

The lipophilicity of [^18^F]**FESCH** was examined by partitioning between *n*-octanol and phosphate-buffered saline (PBS, pH = 7.4) at ambient temperature using the conventional shake-flask method. The radiotracer (65 µL, ~ 8 MBq) was added to a tube containing the *n*-octanol/PBS-mixture (6 mL, 1:1, four-fold determination). The tubes were shaken for 20 min using a mechanical shaker (HS250 basic, IKA Labortechnik GmbH & Co. KG, Staufen, Germany) followed by centrifugation (5500 rpm for 5 min) and separation of the phases. Aliquots were taken from the organic and the aqueous phase (1 mL each) and activity was measured with an automated gamma counter (1480 WIZARD, Fa. Perkin Elmer, Waltham, MA, USA). The distribution coefficient (D_7.4_) was calculated as [activity (cpm/mL) in *n*-octanol]/[activity (cpm/mL) in PBS, pH = 7.4] stated as the decade logarithm (logD_7.4_).

### 4.4. Binding Assay

Membrane homogenates prepared from stably transfected CHO-K1 cells (Chinese hamster ovary cells, clone K1) with the human A_1_R or A_2A_R, provided by Prof. Klotz, University of Würzburg, Würzburg, Germany, were used for the binding experiments. The following radioligands were employed: [^3^H]**ZM241385** for the A_2A_R and [^3^H]**DPCPX** for the A_1_R. For the determination of A_2A_R and A_1_R binding affinity of reference compound, frozen cell suspensions were thawed, homogenized by a 27-gauge needle, and diluted with 50 mM TRIS-HCl buffer (pH = 7.4, 100 mM NaCl, 5 mM MgCl_2_, 1 mM EDTA) containing 1 µU/mL adenosine deaminase (ADA). Membrane suspension was incubated with 1.5 nM [^3^H]**ZM241385** or 1 nM [^3^H]**DPCPX** and various concentrations of the test compound. Non-specific binding was determined by co-incubation with 10 μM **ZM241385** or 1 µM **DPCPX**. The incubation was performed at room temperature for 90 min and terminated by rapid filtration using Whatman GF/B glass-fiber filters, pre-soaked in 0.3% polyethyleneimine and a 48-channel harvester (Biomedical Research and Development Laboratories, Gaithersburg, MD, USA) followed by washing four times with ice-cold TRIS-HCl buffer. Filter-bound radioactivity was quantified by liquid scintillation counting. At least two separate experiments were performed for determination of *K*_i_ values. All data were analyzed with GraphPad Prism, Version 4.1 (GraphPad Inc., La Jolla, CA, USA), according to the Cheng–Prusoff equation.

### 4.5. Animal Experiments

All experimental work including animals was conducted in accordance with the national legislation on the use of animals for research (Tierschutzgesetz (TierSchG), Tierschutz-Versuchstierverordnung (TierSchVersV)) and was approved by the responsible animal care committees of the Free State of Bavaria and the Free State of Saxony (TVV 08/13, 24-9168.11/18/8, June 12th, 2013 and TVV 18/18, DD24.1-5131/446/19, June 20th, 2018; Landesdirektion Sachsen). Female CD-1 mice, 10–12 weeks, were obtained from the Medizinisch-Experimentelles Zentrum at University of Leipzig, Leipzig, Germany.

#### 4.5.1. In Vitro Autoradiography

Cryosections of brains obtained from female CD-1 mice (10–12 weeks) were thawed, dried in a stream of cold air, and pre-incubated in 50 mM TRIS-HCl buffer (pH = 7.4, 100 mM NaCl, 5 mM MgCl_2_, 1 mM EDTA) containing 1 µU/mL adenosine deaminase (ADA) for 15 min at ambient temperature. Afterwards, brain sections were incubated with 0.1–0.2 MBq/mL [^18^F]**FESCH** in buffer for 90 min at room temperature. Non-specific binding was determined in the presence of 1 µM of **FESCH** or **ZM241385**, respectively. Subsequently, the sections were washed twice for 5 min in ice-cold TRIS-HCl buffer, and dipped for 5 s in ice-cold deionized water. The sections were rapidly dried in a stream of cold air before being exposed overnight on an imaging plate (Fujifilm Corporation, Tokyo, Japan). Developed autoradiographs were analyzed in a phosphor imager (HD-CR 35, Duerr NDT GmbH, Bietigheim Bissingen, Germany). The quantification was performed by using 2D-densitometric analysis (AIDA 2.31 software, raytest Isotopenmessgeräte GmbH, Straubenhardt, Germany). Further data analysis was performed with GraphPad Prism, Version 4.1 (GraphPad Inc., La Jolla, CA, USA).

#### 4.5.2. Rotenone-Based and A30P Transgenic Mouse Models of Parkinson’s Disease

##### Animal Housing

Male wild-type C57BL/6JRj mice (Janvier Labs, Le Genest-Saint-Isle, France) and male (Thy1)-*h*[A30P]α-syn transgenic C57BL/6JRj mice (generated according to the protocol by Kahle et al. [43], hereafter “A30P transgenic mice”) were housed at room temperature under a 12:12 h dark:light cycle. Food and water was provided ad libidum.

##### Oral Rotenone Administration

Wild-type C57BL/6JRj mice (12 months) were divided into two groups and treated 5 days a week for 4 months. A 1.2 mm × 60 mm gavage (Unimed, Lausanne, Switzerland) was used to administer 0.01 mL/g animal weight of rotenone (Sigma-Aldrich, Munich, Germany) solution corresponding to a 5 mg/kg dose. Controls were treated only with the vehicle solution (2% carboxymethyl cellulose (Sigma-Aldrich, Munich, Germany) and 1.25% chloroform (Carl Roth, Karlsruhe, Germany)).

#### 4.5.3. Immunofluorescence Staining and Imaging

##### Immunostaining

Cryosections of brains obtained from wild-type C57BL/6JRj mice (16 months, vehicle and rotenone-treated) or A30P transgenic mice (young group = 39 weeks; old group = 71–76 weeks) were immunostained using the free-floating technique and all procedures took place in a well of a 12-well plate. Briefly, brain sections were washed with PBS (pH = 7.4) for 10 min, then blocked with blocking solution (PBS, 0.02% Triton-X and 5% donkey serum) for 1 h, and incubated in the first antibody (1:100, rabbit anti-adenosine receptor A_2A_, ab3461, Abcam, Cambridge, UK) in blocking solution at 4 °C overnight. On the next day, sections were washed in PBS (4 × 15 min) and incubated with the secondary antibody (1:500, donkey Alexa^®^ 555 anti-rabbit, Invitrogen/Thermo Fisher Scientific Inc., Waltham, MA, USA) for three hours at room temperature. Sections were washed in PBS (4 × 15 min), transferred to the slide, and mounted with Mowiol mounting medium (50% PBS, 25% ethylenglycol and 25% glycerol).

##### Microscopy and Image Analysis

Images were obtained with an Apotome.2 fluorescence microscope (Carl Zeiss Microscopy GmbH, Jena, Germany). Mean fluorescence intensity (MFI) in the striatum was measured using Fiji-ImageJ, a free-software for image analysis (ImageJ v1.52p, National Institutes of Health (NIH), Bethesda, MD, USA). MFI values were normalized to background signal.

#### 4.5.4. Small Animal PET/MR Studies

For the time of the experiments, female CD-1 mice (n = 12; age = 10 weeks; weight = 30–35 g) and male C57BL/6JRj mice (control with n = 5; rotenone-treated with n = 7; age = 16 months; weight = 28–35 g) were kept in a dedicated climatic chamber with free access to water and food under a 12:12 h dark:light cycle at a constant temperature of 24 °C. The animals received an injection of [^18^F]**FESCH** into the tail vein (5.0 ± 2.5 MBq for CD-1 mice) or retro-orbital (9.7 ± 1.3 MBq for C57BL/6JRj mice) followed by a 60-min PET/MR scan (Mediso nanoScan®, Budapest, Hungary). Each PET image was corrected for random coincidences, dead time, scatter, and attenuation (AC), based on a whole body (WB) MR scan. The reconstruction parameters for the list mode data were the following: 3D-ordered subset expectation maximization (OSEM), 4 iterations, 6 subsets, energy window = 400–600 keV, coincidence mode = 1–5, ring difference = 81. The mice were positioned prone in a special mouse bed (heated up to 37 °C), with the head fixed to a mouth piece for the anesthetic gas supply with isoflurane in 40% air and 60% oxygen (anesthesia unit: U-410, Agnthos, Lidingö, Sweden; gas blender: MCQ, Rome, Italy). The PET data were collected by a continuous WB scan during the entire investigation. Following the 60-min PET scan, a T1-weighted WB gradient echo sequence (GRE, repetition time = 20 ms, echo time = 6.4 ms) was performed for AC and anatomical orientation. Image registration and evaluation of the region of interest (ROI) were done with PMOD (PMOD Technologies LLC, v. 3.9, Zurich, Switzerland). 

The respective brain regions were identified using the mouse brain atlas template Ma–Benveniste–Mirrione–FDG. The activity data are expressed as mean standardized uptake value (SUV) of the overall ROI.

#### 4.5.5. In Vivo Metabolism Study

The radiotracer [^18^F]**FESCH** (~ 17 MBq in 150 µL isotonic saline) was injected into a female CD-1 mouse via the tail vein. Brain and blood samples were obtained at 15 min p.i., plasma separated by centrifugation (14,000 × *g*, 1 min), and brain homogenized in ~ 1 mL isotonic saline on ice (10 strokes of a polytetrafluoroethylene (Teflon^®^) plunger at 1000 rpm in a borosilicate glass cylinder; Potter S Homogenizer, B. Braun Melsungen AG, Melsungen, Germany).

Two consecutive extractions were performed as duplicate (plasma) or triplicate (brain) determinations. Plasma (50 µL) and brain samples (250 µL) were added to an ice-cold acetone/water mixture (4:1; plasma or brain sample/organic solvent = 1:4). The samples were vortexed for 1 min, incubated on ice for 10 min (first extraction) or 5 min (second extraction) and centrifuged at 10,000 rpm for 5 min. Supernatants were collected and the precipitates were re-dissolved in 100 µL of the ice-cold acetone/water mixture (4:1) for the second extraction. Activity of aliquots from supernatants of each extraction step and of the precipitates was quantified using an automated gamma counter (1480 WIZARD, Fa. Perkin Elmer). The supernatants from both extractions were combined, concentrated at 70 °C under argon stream, and analyzed by radio-HPLC (gradient mode, see General Information). By this protocol, [^18^F]**FESCH** was quantitatively extracted from the biological material as proven by in vitro incubation of the radiotracer in pig plasma.

## References

[1] Gerlach M., Reichmann H., Riederer P. (2007). Die Parkinson-Krankheit: Grundlagen, Klinik, Therapie.

[2] De Lera Ruiz M., Lim Y.-H., Zheng J. (2014). Adenosine A_2A_ receptor as a drug discovery target. J. Med. Chem..

[3] Jorg M., Scammells P.J., Capuano B. (2014). The dopamine D_2_ and adenosine A_2A_ receptors: Past, present and future trends for the treatment of Parkinson’s disease. Curr. Med. Chem..

[4] Ferre S., von Euler G., Johansson B., Fredholm B.B., Fuxe K. (1991). Stimulation of high-affinity adenosine A_2_ receptors decreases the affinity of dopamine D_2_ receptors in rat striatal membranes. Proc. Natl. Acad. Sci. USA.

[5] Ferré S., Bonaventura J., Tomasi D., Navarro G., Moreno E., Cortés A., Lluís C., Casadó V., Volkow N.D. (2016). Allosteric mechanisms within the adenosine A_2A_–dopamine D_2_ receptor heterotetramer. Neuropharmacology.

[6] Nazario L.R., da Silva R.S., Bonan C.D. (2017). Targeting adenosine signaling in Parkinson’s disease: From pharmacological to non-pharmacological approaches. Front. Neurosci..

[7] LeWitt P.A., Guttman M., Tetrud J.W., Tuite P.J., Mori A., Chaikin P., Sussman N.M., Group U.S.S. (2008). Adenosine A_2A_ receptor antagonist istradefylline (KW-6002) reduces “off” time in Parkinson’s disease: A double-blind, randomized, multicenter clinical trial (6002-US-005). Ann. Neurol..

[8] Hauser R.A., Cantillon M., Pourcher E., Micheli F., Mok V., Onofrj M., Huyck S., Wolski K. (2011). Preladenant in patients with Parkinson’s disease and motor fluctuations: A phase 2, double-blind, randomised trial. Lancet Neurol..

[9] Hauser R.A., Olanow C.W., Kieburtz K.D., Pourcher E., Docu-Axelerad A., Lew M., Kozyolkin O., Neale A., Resburg C., Meya U. (2014). Tozadenant (SYN115) in patients with Parkinson’s disease who have motor fluctuations on levodopa: A phase 2b, double-blind, randomised trial. Lancet Neurol..

[10] Dungo R., Deeks E.D. (2013). Istradefylline: First global approval. Drugs.

[11] Villar-Menéndez I., Porta S., Buira S.P., Pereira-Veiga T., Díaz-Sánchez S., Albasanz J.L., Ferrer I., Martín M., Barrachina M. (2014). Increased striatal adenosine A_2A_ receptor levels is an early event in Parkinson’s disease-related pathology and it is potentially regulated by miR-34b. Neurobiol. Dis..

[12] Khanapur S., Waarde A.V., Ishiwata K., Leenders K.L., Dierckx R.A.J.O., Elsinga P.H. (2014). Adenosine A_2A_ receptor antagonists as positron emission tomography (PET) tracers. Curr. Med. Chem..

[13] Ramlackhansingh A.F., Bose S.K., Ahmed I., Turkheimer F.E., Pavese N., Brooks D.J. (2011). Adenosine 2A receptor availability in dyskinetic and nondyskinetic patients with Parkinson disease. Neurology.

[14] Barret O., Hannestad J., Alagille D., Vala C., Tavares A., Papin C., Morley T., Fowles K., Lee H., Seibyl J. (2014). Adenosine 2A receptor occupancy by tozadenant and preladenant in rhesus monkeys. J. Nucl. Med..

[15] Barret O., Hannestad J., Vala C., Alagille D., Tavares A., Laruelle M., Jennings D., Marek K., Russell D., Seibyl J. (2015). Characterization in humans of ^18^F-MNI-444, a novel PET radiotracer for brain adenosine A_2A_ receptors. J. Nucl. Med..

[16] Chen J.-F., Eltzschig H.K., Fredholm B.B. (2013). Adenosine receptors as drug targets—What are the challenges?. Nat. Rev. Drug Discov..

[17] Svenningsson P., Hall H., Sedvall G., Fredholm B.B. (1997). Distribution of adenosine receptors in the postmortem human brain: An extended autoradiographic study. Synapse.

[18] Shinkre B.A., Kumar T.S., Gao Z.-G., Deflorian F., Jacobson K.A., Trenkle W.C. (2010). Synthesis and evaluation of 1,2,4-triazolo[1,5-*c*]pyrimidine derivatives as A_2A_ receptor-selective antagonists. Bioorg. Med. Chem. Lett..

[19] Bhattacharjee A.K., Lang L., Jacobson O., Shinkre B., Ma Y., Niu G., Trenkle W.C., Jacobson K.A., Chen X., Kiesewetter D.O. (2011). Striatal adenosine A_2A_ receptor-mediated positron emission tomographic imaging in 6-hydroxydopamine-lesioned rats using [^18^F]-MRS5425. Nucl. Med. Biol..

[20] Khanapur S., Paul S., Shah A., Vatakuti S., Koole M.J.B., Zijlma R., Dierckx R.A.J.O., Luurtsema G., Garg P., van Waarde A. (2014). Development of [^18^F]-labeled pyrazolo[4,3-*e*]-1,2,4- triazolo[1,5-*c*]pyrimidine (SCH442416) analogs for the imaging of cerebral adenosine A_2A_ receptors with positron emission tomography. J. Med. Chem..

[21] Khanapur S., van Waarde A., Dierckx R.A., Elsinga P.H., Koole M.J. (2017). Preclinical evaluation and quantification of ^18^F-fluoroethyl and ^18^F-fluoropropyl analogs of SCH442416 as radioligands for PET imaging of the adenosine A_2A_ receptor in rat brain. J. Nucl. Med..

[22] Pan-Montojo F., Anichtchik O., Dening Y., Knels L., Pursche S., Jung R., Jackson S., Gille G., Spillantini M.G., Reichmann H. (2010). Progression of Parkinson’s disease pathology is reproduced by intragastric administration of rotenone in mice. PLoS ONE.

[23] Arnhold M., Dening Y., Chopin M., Arévalo E., Schwarz M., Reichmann H., Gille G., Funk R.H.W., Pan-Montojo F. (2016). Changes in the sympathetic innervation of the gut in rotenone-treated mice as possible early biomarker for Parkinson’s disease. Clin. Auton. Res..

[24] Pan-Montojo F., Schwarz M., Winkler C., Arnhold M., O’Sullivan G.A., Pal A., Said J., Marsico G., Verbavatz J.M., Rodrigo-Angulo M. (2012). Environmental toxins trigger PD-like progression via increased alpha-synuclein release from enteric neurons in mice. Sci. Rep..

[25] Moon Y., Lee K.H., Park J.-H., Geum D., Kim K. (2005). Mitochondrial membrane depolarization and the selective death of dopaminergic neurons by rotenone: Protective effect of coenzyme Q10. J. Neurochem..

[26] Betarbet R., Sherer T.B., MacKenzie G., Garcia-Osuna M., Panov A.V., Greenamyre J.T. (2000). Chronic systemic pesticide exposure reproduces features of Parkinson’s disease. Nat. Neurosci..

[27] Baraldi P.G., Cacciari B., Romagnoli R., Spalluto G., Monopoli A., Ongini E., Varani K., Borea P.A. (2002). 7-Substituted 5-amino-2-(2-furyl)pyrazolo[4,3-*e*]-1,2,4-triazolo[1,5-*c*]pyrimidines as A_2A_ adenosine receptor antagonists:  A study on the importance of modifications at the side chain on the activity and solubility. J. Med. Chem..

[28] Damont A., Hinnen F., Kuhnast B., Schöllhorn-Peyronneau M.-A., James M., Luus C., Tavitian B., Kassiou M., Dollé F. (2008). Radiosynthesis of [^18^F]DPA-714, a selective radioligand for imaging the translocator protein (18 kDa) with PET. J. Label. Compd. Rad..

[29] Schröder S., Lai T.H., Kranz M., Toussaint M., Shang Q., Dukic-Stefanovic S., Pan-Montojo F., Brust P. (2019). Investigation of [^18^F]FESCH for PET imaging of the adenosine A_2A_ receptor in a rotenone-based mouse model of Parkinson´s disease and development of a two-step one-pot radiolabeling strategy. J. Label. Compd. Rad..

[30] Tavares A.A.S., Lewsey J., Dewar D., Pimlott S.L. (2012). Radiotracer properties determined by high performance liquid chromatography: A potential tool for brain radiotracer discovery. Nucl. Med. Biol..

[31] Clark D.E. (2003). *In silico* prediction of blood–brain barrier permeation. Drug Discov. Today.

[32] Waterhouse R.N. (2003). Determination of lipophilicity and its use as a predictor of blood–brain barrier penetration of molecular imaging agents. Mol. Imaging Biol..

[33] Sihver W., Schulze A., Wutz W., Stusgen S., Olsson R.A., Bier D., Holschbach M.H. (2009). Autoradiographic comparison of *in vitro* binding characteristics of various tritiated adenosine A_2A_ receptor ligands in rat, mouse and pig brain and first *ex vivo* results. Eur. J. Pharmacol..

[34] Schröder S., Wenzel B., Deuther-Conrad W., Teodoro R., Kranz M., Scheunemann M., Egerland U., Höfgen N., Briel D., Steinbach J. (2018). Investigation of an ^18^F-labelled imidazopyridotriazine for molecular imaging of cyclic nucleotide phosphodiesterase 2A. Molecules.

[35] Liu J., Wenzel B., Dukic-Stefanovic S., Teodoro R., Ludwig F.-A., Deuther-Conrad W., Schröder S., Chezal J.-M., Moreau E., Brust P. (2016). Development of a new radiofluorinated quinoline analog for PET imaging of phosphodiesterase 5 (PDE5) in brain. Pharmaceuticals.

[36] Zoghbi S.S., Shetty H.U., Ichise M., Fujita M., Imaizumi M., Liow J.-S., Shah J., Musachio J.L., Pike V.W., Innis R.B. (2006). PET imaging of the dopamine transporter with [^18^F]FECNT: A polar radiometabolite confounds brain radioligand measurements. J. Nucl. Med..

[37] Evens N., Vandeputte C., Muccioli G.G., Lambert D.M., Baekelandt V., Verbruggen A.M., Debyser Z., Van Laere K., Bormans G.M. (2011). Synthesis, *in vitro* and *in vivo* evaluation of fluorine-18 labelled FE-GW405833 as a PET tracer for type 2 cannabinoid receptor imaging. Bioorgan. Med. Chem..

[38] Lear J.L., Ackermann R.F. (1990). Evaluation of radiolabeled acetate and fluoroacetate as potential tracers of cerebral oxidative metabolism. Metab. Brain Dis..

[39] Mori T., Sun L.-Q., Kobayashi M., Kiyono Y., Okazawa H., Furukawa T., Kawashima H., Welch M.J., Fujibayashi Y. (2009). Preparation and evaluation of ethyl[^18^F]fluoroacetate as a proradiotracer of [^18^F]fluoroacetate for the measurement of glial metabolism by PET. Nucl. Med. Biol..

[40] Muir D., Berl S., Clarke D.D. (1986). Acetate and fluoroacetate as possible markers for glial metabolism *in vivo*. Brain Res..

[41] Ponde D.E., Dence C.S., Oyama N., Kim J., Tai Y.-C., Laforest R., Siegel B.A., Welch M.J. (2007). ^18^F-Fluoroacetate: A potential acetate analog for prostate tumor imaging—*In vivo* evaluation of ^18^F-fluoroacetate versus ^11^C-acetate. J. Nucl. Med..

[42] Peng Q., Zhong S., Tan Y., Zeng W., Wang J., Cheng C., Yang X., Wu Y., Cao X., Xu Y. (2019). The rodent models of dyskinesia and their behavioral assessment. Front. Neurol..

[43] Kahle P.J., Neumann M., Ozmen L., Müller V., Jacobsen H., Schindzielorz A., Okochi M., Leimer U., van der Putten H., Probst A. (2000). Subcellular localization of wild-type and Parkinson’s disease-associated mutant α-synuclein in human and transgenic mouse brain. J. Neurosci..

[44] Wagner J., Ryazanov S., Leonov A., Levin J., Shi S., Schmidt F., Prix C., Pan-Montojo F., Bertsch U., Mitteregger-Kretzschmar G. (2013). Anle138b: A novel oligomer modulator for disease-modifying therapy of neurodegenerative diseases such as prion and Parkinson’s disease. Acta Neuropathol..

[45] Kuchar M., Mamat C. (2015). Methods to increase the metabolic stability of ^18^F-radiotracers. Molecules.

